# Estimation of Nanoporous Au Young’s Modulus from Serial Block Face-SEM 3D-Characterisation

**DOI:** 10.3390/ma15103644

**Published:** 2022-05-19

**Authors:** Michele Brun, Elisa Sogne, Andrea Falqui, Federico Scaglione, Paola Rizzi, Francesco Delogu, Giorgio Pia

**Affiliations:** 1 Dipartimento di Ingegneria Meccanica, Chimica e dei Materiali, Università degli Studi di Cagliari, Piazza d’Armi, 09123 Cagliari, Italy; francesco.delogu@unica.it; 2 NABLA Lab, Biological and Environmental Sciences and Engineering (BESE) Division, King Abdullah University of Science and Technology (KAUST), Thuwal 23955-6900, Saudi Arabia; eli.sogne@gmail.com; 3 Dipartimento di Fisica “Aldo Pontremoli”, Università degli Studi di Milano, Via Celoria 16, 20133 Milan, Italy; andrea.falqui@unimi.it; 4 Dipartimento di Chimica and NIS, Università di Torino, Via Giuria 7, 10125 Torino, Italy; federico.scaglione@unito.it (F.S.); paola.rizzi@unito.it (P.R.)

**Keywords:** nanoporous metals, mechanical properties, serial block face-SEM, multiscale analysis, digital image correlation

## Abstract

Nanoporous Au has been subjected to serial block face-scanning electron microscopy (SBF-SEM) 3D-characterisation. Corresponding sections have been digitalized and used to evaluate the associated mechanical properties. Our investigation demonstrates that the sample is homogeneous and isotropic. The effective Young’s modulus estimated by an analytical multiscale approach agrees remarkably well with the values stated in the literature.

## 1. Introduction

Nanoporous (NP) Au displays a three-dimensional open-cell structure with relatively stocky ligaments joining at irregularly shaped nodes [1]. The structure characterises the whole class of materials and exhibits significant self-similarity over length scales ranging from 5 to 200 nm. Its global and local morphologies are sensitive to the experimental conditions underlying preparation, typically based on the selective dissolution of less-noble elements from the starting alloy [1]. The rearrangement of solid–liquid interfaces by surface diffusion results in a highly porous, bi-continuous network with physical-chemical properties of interest for several applications [2,3,4].

Their mechanical behaviour in particular has drawn attention from different areas of science and engineering. Strongly affected by porosity degree and characteristic lengths, their mechanical properties still need accurate scrutiny to reveal their connection with the structure.

This is precisely what we do in the present work. Serial Block Face-Scanning Electron Microscopy (SBF-SEM) was used for 3D reconstructing a very large NP Au volume, using a technique which allows the 3D-characterisation of volume as big as many tens of cubic microns, while keeping the typical x-y lateral resolution of a SEM (down to very few nanometres) and the z-one defined by the cutting step used in the experiments [5]. Furthermore, the majority of the analyses derive the macroscopic properties from phenomenological models based on internal morphological parameters, such as solid phase volume fraction, characteristic length and thickness of ligament size [6,7] or connectivity [8], while numerical computations are generally based on Finite Element simulations on representative microstructures, which are assumed to be periodic or numerically randomized [8], in particular with gyroidal [9] or spinodal [10] microgeometries. Alternatively, computationally expensive Molecular Dynamics simulations have been proposed [11]. More refined descriptions of the microstructure require ad hoc approaches [12], which are needed to reduce the computational cost [10].

Here, we propose an alternative methodology capable of deriving macroscopic mechanical properties from SEM data, which have been digitalized in order to produce a regular voxel grid, with each voxel characterised by solid or void phase. Then, a semi-analytical multiscale model is implemented with the ability to describe the effect of the complicated internal morphology of a very large sample on the macroscopic mechanical properties. 

Specifically, we have simulated the bending behaviour of a NP Au beam with the same homogeneity and isotropy as the sample characterised experimentally. We noted that the estimated Young’s modulus was in excellent agreement with the experimental value.

## 2. Materials and Methods

An Au_20_Cu_48_Ag_7_Pd_5_Si_20_ master alloy was prepared by arc melting the elements with 99.99% purity. Amorphous ribbons about 20 mm thick and 2 mm wide were obtained by melt spinning. The NP Au samples were prepared by dealloying amorphous ribbons in free corrosion conditions at 70 °C in an aqueous solution of 10 M HNO_3_ and 0.5 M HF for 4 h [13,14].

The NP Au ribbon was infiltrated and embedded in epoxy resin cured at 60 °C for 48 h. Resin blocks were mounted on an aluminium specimen pin using acrylic glue and silver paint was used to electrically ground the edges of the block to the aluminium pin. Then, the entire sample was sputter-coated with 25 nm layer of Au by a high-resolution sputter coater (Cressington 208HR, Cressington Scientific Instruments Ltd., Watford, UK). SBF-SEM imaging was performed using a Gatan 3View SBF system, mounted on a FEI Quanta FEG 200 microscope. The latter was operated at an acceleration voltage of 3 kV, with a beam current of 21 pA and an in-chamber pressure of 10 Pa of water vapor. Samples were imaged using the backscattered electrons signal, with a field of view of 7.2 × 7.2 µm^2^ and a resolution of 1200 × 1200 pixel. The corresponding x-y-z voxel size was 6 × 6 × 50 nm^3^, the last value corresponding to the thickness used for the SEM cutting interval. More details on the SBF-SEM technique can be found in [15].

## 3. Results and Discussion

**Figure 1:**
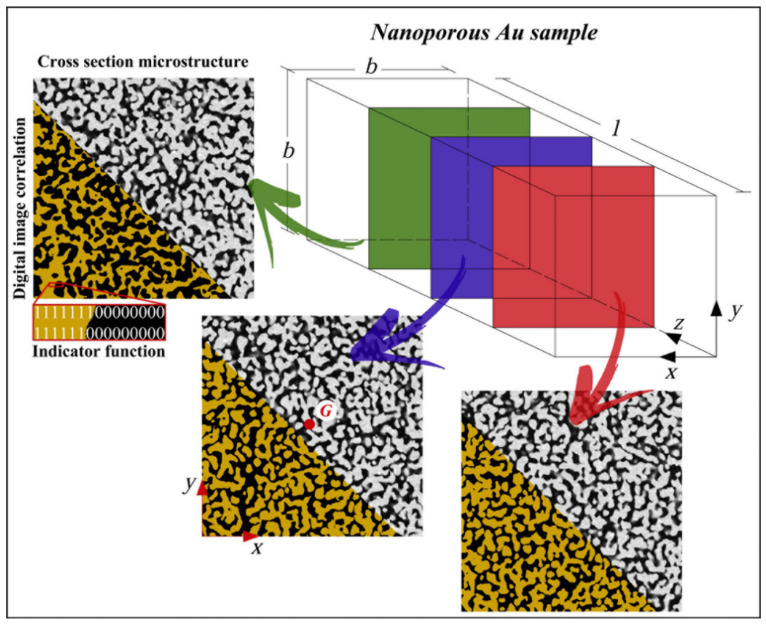
The NP Au sample with sides b=7.2 µm and l=12.8 µm. The 256 sections obtained by SBF-SEM have been transformed by digital image correlation in a voxel structure. Gold and black colors indicate solid and void phases. The indicator function $\theta_{ij}^{\left(s\right)}$ is equal to 1 for solid and 0 for void phases.

Each SBF-SEM 3D section s out of the 256 available was divided in a 1200 × 1200 grid of voxels. A voxel indicator function $\theta_{ij}^{\left(s\right)}$ was used to associate the 1 or 0 values, respectively, to solid and void voxels. Value 1 was assigned to voxels with a greyscale colour less than 50%, and value 0 was assigned otherwise. Filtering values of 25% and 75% gave negligible variations of the results. The indices i and j indicate the voxel position, respectively, along the N rows and N columns in each section. As shown in Figure 1, this allowed producing digitalized copies of the SBF-SEM 3D sections.

The volume fraction of a solid in each section s is

(1) $$\phi^{\left(s\right)}=\frac{\sum_{i,j=1}^{N}\theta_{ij}^{\left(s\right)}}{N^{2}}\;$$

where the longitudinal coordinate $z=l\left(\frac{s}{256}\right).$

The normalised centre of mass has the coordinates

(2) $$x_{G}^{\left(s\right)}=\frac{1}{N^{2}}\sum_{i,j=1}^{N}\theta_{ij}^{\left(s\right)}\left(j-\frac{1}{2}\right)$$

(3) $$y_{G}^{\left(s\right)}=\frac{1}{N^{2}}\sum_{i,j=1}^{N}\theta_{ij}^{\left(s\right)}\left(i-\frac{1}{2}\right)$$

Moments of inertia about central axes, normalized with respect to a solid cross-section, are

(4) $$I_{x}{}^{\left(s\right)}=\frac{12}{N^{4}}\sum_{i,j=1}^{N}\left[\theta_{ij}^{\left(s\right)}\left(\frac{1}{12}+{\left(j-\frac{1}{2}-x_{G}^{\left(s\right)}\right)}^{2}\right)\right]\;I_{y}{}^{\left(s\right)}=\frac{12}{N^{4}}\sum_{i,j=1}^{N}\left[\theta_{ij}^{\left(s\right)}\left(\frac{1}{12}+{\left(i-\frac{1}{2}-y_{G}^{\left(s\right)}\right)}^{2}\right)\right]\;I_{xy}{}^{\left(s\right)}=\frac{12}{N^{4}}\sum_{i,j=1}^{N}\left[\theta_{ij}^{\left(s\right)}\left(j-\frac{1}{2}-x_{G}^{\left(s\right)}\right)\cdot \left(i-\frac{1}{2}-y_{G}^{\left(s\right)}\right)\right]$$

**Figure 2:**
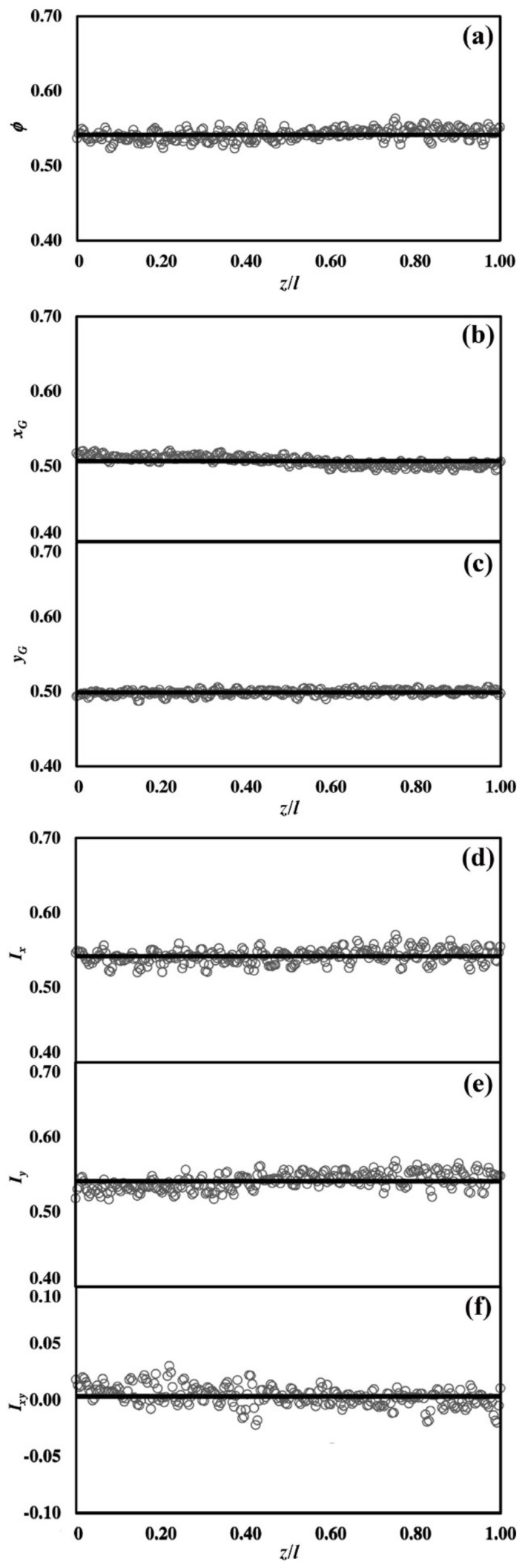
The NP (**a**) Volume fraction ϕ, (**b**,**c**) centre of mass position $\left(x_{G},y_{G}\right)$, and (**d**–**f**) moments of inertia $\left(I_{x},I_{y},I_{xy}\right)$ along the longitudinal direction z/l. The average values $\bar{\phi }=0.5416$, $\left({\bar{x}}_{G},{\bar{y}}_{G}\right)=\left(0.5069,\;0.4988\right)$ and $\left({\bar{I}}_{x},{\bar{I}}_{y},{\bar{I}}_{xy}\right)=\left(0.5420,\;0.5409,\;0.0032\right)\;$ are shown as black continuous lines.

The $\phi^{\left(s\right)}$, $x_{G}^{\left(s\right)}$, $y_{G}^{\left(s\right)}$, $I_{x}{}^{\left(s\right)}$, $I_{y}{}^{\left(s\right)}$ and $I_{xy}{}^{\left(s\right)}$ estimates obtained for the different sections s are shown in Figure 2. The average values ${\bar{x}}_{G}≃{\bar{y}}_{G}≃0.5$, ${\bar{I}}_{x}≃{\bar{I}}_{y}$ and ${\bar{I}}_{xy}≃0$ emphasize the isotropic distribution of the solid phase in each section. The $\phi^{\left(s\right)}$ averages around 0.54, whereas the ${\bar{I}}_{x}$ and ${\bar{I}}_{y}$, which measure the bending stiffness, average to 0.54 [16].

With their low dispersion degree around the average values, the estimates point out the remarkable homogeneity and isotropy of the NP Au structure along the longitudinal sample axis, z. These results confirm the anisotropy estimates given by Mangipadi, obtained by analysing the directional pair correlation function on samples of much smaller dimensions [10]. These results can be ascribed to the fabrication method capability of inducing similar dealloying behaviour, even relatively far from sample free surfaces. Accordingly, it can be expected that the effective Young’s modulus depends on the intrinsic NP Au structure and properties, and not on the sample size.

The effective Young’s modulus was estimated through a multiscale approach inspired to fractal modelling [17,18]. To this aim, we divided the sample volume in square cuboids having $N_{1}$ elements along the x and y sides and $N_{2}$ along the z one and, thus, containing a total of $N_{1}\times N_{1}\times N_{2}$ elements. Calculations were initially carried out for the case of elements corresponding to voxels and square cuboids involving 3 × 3 × 2 voxels. Accordingly, each square cuboid consisted of two planes, parallel to the Cartesian (x,y) plane, containing 3 × 3 voxels superposed along the z direction. The effective Young’s modulus of the square cuboid was calculated using the combination of springs in parallel on the plane and springs in series along the longitudinal direction [16]. The Young’s modulus of each voxel, ${\tilde{E}}_{ij}^{\left(k\right)}$, was set equal to the one of bulk Au, E, for solid voxels or to 0 for void voxels. Therefore, a specific effective Young’s modulus was associated with any given square cuboid.

The calculations described above were repeated with square cuboids containing 4 × 4 × 2 elements. This time, however, each element corresponded to one of the square cuboids containing the 3 × 3 × 2 voxels previously considered. The Young’s modulus of each element was set equal to the effective Young’s modulus of the corresponding square cuboid, as calculated in the first iteration.

The calculation scheme was implemented for the other three iterations, every time involving a scale up of the reference square cuboid as described above. In particular, square cuboids were made up of 4 × 4 × 4, 5 × 5 × 4 and, again, 5 × 5 × 4 elements were considered in the third, fourth and fifth iterations.

In each iteration m, the longitudinal Young’s modulus ${\tilde{E}}_{z,m}$ was calculated using the equation

(5) $${\tilde{E}}_{z,m}=\frac{N_{2,m}}{N_{1,m}^{2}}{\left[\sum_{k=1}^{N_{2,m}}\left(\frac{1}{\sum_{i,j=1}^{N_{1,m}}{\tilde{E}}_{ij}^{\left(k\right)}\left(m\right)}\right)\right]}^{-1},\;$$

where, ${\tilde{E}}_{ij}^{\left(k\right)}\left(m\right)$ is the effective Young’s modulus of the element identified by indices i and j, and $N_{1,m}$ and $N_{2,m}$ are the number of elements determining the volume of cuboids at iteration m.

The relative effective Young’s modulus along the z direction was set equal to

(6) $$E_{z}=\frac{{\tilde{E}}_{z,5}}{E}.\;$$

Similarly, the effective Young’s moduli along transversal directions, $E_{x}$ and $E_{y}$, were calculated for each section s using the equations

(7) $${\tilde{E}}_{x,m}^{\left(s\right)}={\left[\sum_{i=1}^{N_{1,m}}\left(\frac{1}{\sum_{j=1}^{N_{1,m}}{\tilde{E}}_{ij}^{\left(s\right)}\left(m\right)}\right)\right]}^{-1},\;$$

(8) $${\tilde{E}}_{y,m}^{\left(s\right)}={\left[\sum_{j=1}^{N_{1,m}}\left(\frac{1}{\sum_{i=1}^{N_{1,m}}{\tilde{E}}_{ij}^{\left(s\right)}\left(m\right)}\right)\right]}^{-1}.\;$$

**Figure 3:**
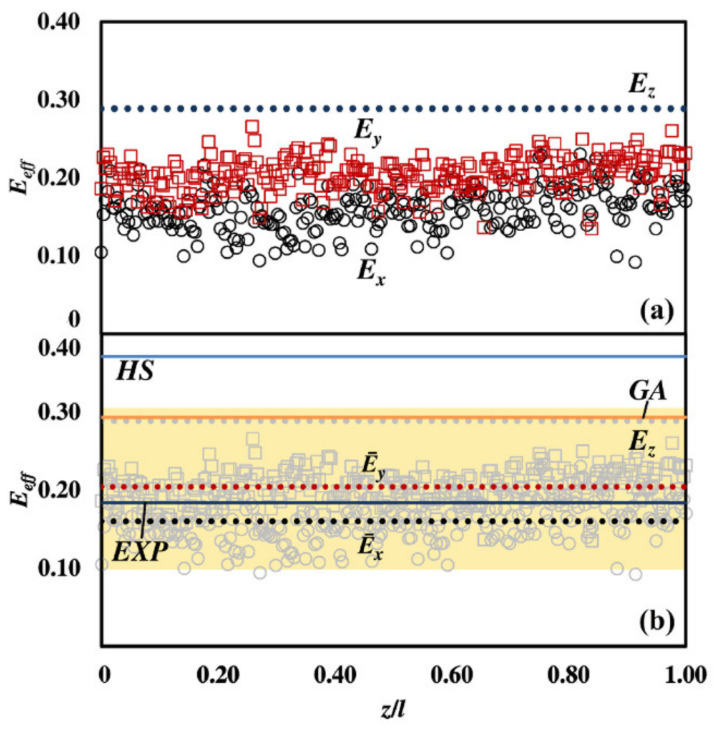
The NP relative effective longitudinal $E_{z}$ and transverse $E_{x}$, $E_{y}$ Young’s moduli. (**a**) $E_{z}$ for the sample and $E_{x}$ (s), $E_{y}\left(s\right)$ in each section s. (**b**) $E_{z}$ and the average values ${\bar{E}}_{x}$ and ${\bar{E}}_{y}$ versus the Hashin-Shtrikman (HS) upper bound, the Gibson and Ashby (GA) and the exponential (EXP) estimates. The yellow region indicates literature data form [19] and Antonoiu [7] on different classes of NP Au and NP Pt samples with relative density $\bar{\phi }$ from 0.5 to 0.6.

The corresponding relative effective Young’s moduli were computed as

(9) $$E_{x}=\frac{{\tilde{E}}_{x,5}}{E},\;$$

(10) $$E_{y}=\frac{{\tilde{E}}_{y,5}}{E},\;$$

The effective Young’s modulus was also estimated with the Hashin–Shtrikman (HS) upper bound [20,21]

(11) $${\tilde{E}}_{HS}=E\frac{\bar{\phi }}{2-\bar{\phi }}\;,\;$$

the phenomenological exponential relation (EXP) [21]

(12) $${\tilde{E}}_{EXP}=E\;\exp\left[\frac{-2\left(1-\bar{\phi }\right)}{\bar{\phi }}\right],\;$$

and the simplified Gibson and Ashby (GA) model [22]

(13) $${\tilde{E}}_{GA}=E{\bar{\phi }}^{2}$$

Additional estimates obtained on the basis of structural features were also considered [6].

The results obtained are summarized in Figure 3. As evident from Figure 3a, the relative longitudinal Young’s modulus $E_{z}$ is equal to 0.289, while the transverse Young’s moduli ${\bar{E}}_{x}$ and ${\bar{E}}_{y}$ have average values of about 0.170 and 0.205, respectively. The difference between the longitudinal and transverse estimates can be ascribed to the fact that individual sections can comprise portions of solids apparently disconnected from each other, which drastically reduces the Young’s modulus. In any case, data in Figure 3b clearly show the excellent agreement between our estimates for the longitudinal modulus $E_{z}$ and the Gibson and Ashby’s one, and between our estimates for the transverse moduli ${\bar{E}}_{x}$ and ${\bar{E}}_{y}$ and the phenomenological exponential model. Moreover, our estimates also compare remarkably well with those obtained taking into account structural features [19].

## 4. Conclusions

Experimental, analytical and numerical methods have been combined to estimate the effective Young’s modulus of NP Au fabricated by de-alloying. The image analysis procedure shows that the remarkably large NP Au sample has highly homogeneous and nearly isotropic morphology. The semi-analytical Young’s modulus estimates agree well with experimental and predicted values reported in the literature. The proposed approach proves to be a valuable tool to better understand the correlation between the morphology of large NP Au samples and effective properties. The simplified analytical outcomes have been proved to be sufficiently accurate, but the combined approach proposed here is prone to the implementation of more refined numerical homogenization schemes. Nowadays, these are limited by the difficulties and the computational costs associated with the discretisation of the geometry at the resolution scale considered here.
